# Mathematical modeling and multivariate analysis applied earliest soybean harvest associated drying and storage conditions and influences on physicochemical grain quality

**DOI:** 10.1038/s41598-021-02724-y

**Published:** 2021-12-02

**Authors:** Roney Eloy Lima, Paulo Carteri Coradi, Marcela Trojahn Nunes, Sabrina Dalla Corte Bellochio, Newiton da Silva Timm, Camila Fontoura Nunes, Letícia de Oliveira Carneiro, Paulo Eduardo Teodoro, Carlos Campabadal

**Affiliations:** 1 Center of Rural Science, Department of Postgraduate Agricultural Engineering, Federal University of Santa Maria, Avenue Roraima, 1000, Camobi, Santa Maria, RS 97105900 Brazil; 2 Laboratory Postharvest, Department of Agricultural Engineering, Campus Cachoeira do Sul, Federal University of Santa Maria, Avenue Taufik Germano, 3013 - Passo D’Areia, Cachoeira do Sul, RS 96503-205 Brazil; 3 Department of Agronomy, Campus de Chapadão do Sul, Federal University of Mato Grosso do Sul, Chapadão do Sul, MS 79560-000 Brazil; 4 Grain Science and Industry, International Grain Program, Kansas State University, Manhattan, KS 66506 USA

**Keywords:** Environmental sciences, Chemistry, Engineering, Mathematics and computing

## Abstract

Anticipating the harvest period of soybean crops can impact on the post-harvest processes. This study aimed to evaluate early soybean harvest associated drying and storage conditions on the physicochemical soybean quality using of mathematical modeling and multivariate analysis. The soybeans were harvested with a moisture content of 18 and 23% (d.b.) and subjected to drying in a continuous dryer at 80, 100, and 120 °C. The drying kinetics and volumetric shrinkage modeling were evaluated. Posteriorly, the soybean was stored at different packages and temperatures for 8 months to evaluate the physicochemical properties. After standardizing the variables, the data were submitted to cluster analysis. For this, we use Euclidean distance and Ward's hierarchical method. Then defining the groups, we constructed a graph containing the dispersion of the values of the variables and their respective Pearson correlations for each group. The mathematical models proved suitable to describe the drying kinetics. Besides, the effective diffusivity obtained was 4.9 × 10^–10^ m^2^ s^−1^ promoting a volumetric shrinkage of the grains and influencing the reduction of physicochemical quality. It was observed that soybean harvested at 23% moisture, dried at 80 °C, and stored at a temperature below 23 °C maintained its oil content (25.89%), crude protein (35.69%), and lipid acidity (5.54 mL). In addition, it is to note that these correlations' magnitude was substantially more remarkable for the treatments allocated to the G2 group. Furthermore, the electrical conductivity was negatively correlated with all the physicochemical variables evaluated. Besides this, the correlation between crude protein and oil yield was positive and of high magnitude, regardless of the group formed. In conclusion, the early harvest of soybeans reduced losses in the field and increased the grain flow on the storage units. The low-temperature drying and the use of packaging technology close to environmental temperatures conserved the grain quality.

## Introduction

Soybean accounts for approximately 90% of vegetable oil production and more than 80% of biodiesel production^1^. To store and sell soybeans, moisture content should not exceed 14%, which can be reduced to 12%, improving the quality of storage^2^. However, soybean drying when it is not properly controlled and handled can cause physical and latent damage, which may be aggravated in the following stages of storage^3–6^.

The anticipation of the soybean harvest period can impact the post-harvest processes. Thus, early harvesting of soybeans with higher moisture content can reduce adverse effects of weather and climate conditions. In addition, the completion of the harvest period will be possible to manage the soybean batches to improve post-harvest operations and reduce losses in these stages.

It should be noted that on the drying of the grain there are simultaneous heat and mass transfer. Thus, the water is moving in the grains by the liquid diffusion process at drying temperature below 100 °C. In this case, the vaporization of the water takes place on the grain surface. However, when the temperature of the air-drying is above 100 °C, there is usually a vapor diffusion process^7–9^. The drying provides water loss, which may cause damage to the cellular structure of the product; this leads to changes in shape and a decrease in its dimensions^10–12^. However, the shrinkage of plant products during drying is not only linked to water content; it depends also on the drying conditions, shape, and size of the product.

The understanding of the heat and mass transfer process in the drying process implies the decision-making of dryer projects and in the grain mass management during the drying operation^13^. The air temperature and product flow must be monitored during drying, as the variation of these parameters will interfere with the drying time and how the water diffusivity and vaporization of the grains can change the physical and chemical characteristics of the grains, reducing their quality^14–16^.

Under conditions of drying air temperatures above 40 °C, physical damage and reduced physicochemical quality are observed in soybean. At elevated temperatures (> 80 °C), the protein and lipid content can decrease by up to 0.5% and 0.43%, respectively, and the acidity content can increase by up to 0.23 mg KOH/g. The use of mathematical modeling of drying is an alternative to verify which are the best operating conditions and viability of the drying system^17–21^.

Soybean production takes place at specific times of the year, depending on the region. Therefore, for the processing industries to operate all year round, soybeans must be properly stored to supply industrial demand. The storage environment determines the activity of all biotic components in the system, which leads to safe storage or product loss^22–24^. During storage, changes also occur in the physicochemical and technological properties of soybean. The changes are related to the storage time, associated with the temperature and moisture content of the soybean. In addition to the effects caused by the storage conditions, some changes in the soybean may also come from the harvest period and drying conditions used, worsening in storage.

To minimize the effects of drying and storage operations, it is suggested to manage the soybean batches after harvest. As a hypothesis, soybeans harvested in advance, with moisture contents close to 23% (d.b.) would not compromise the flow of batches in the storage units. Thus, drying can be carried out slowly, with a temperature below 100 °C, which would help in the conservation of soybeans during storage at a temperature below 23 °C. The anticipation of the harvest it could be increasing the time for crop rotation in the field, reduce investments with drying and storage structures.

Multivariate analysis has been applied in several studies in the area of drying and grain storage when there is greater experimental complexity^2,6,9^. Due to a large number of treatments in researches in this area, the analysis of principal components and correlations allow verifying the interrelationship of these treatments with the variables evaluated clearly, making it possible to better explore these results. Depending on the experimental conditions involved in this study, it is suggested to apply the technique to verify the groupings of factors and correlations of quantifiable and qualitative variables for a better conclusion. The objective of the study was to evaluate early soybean harvest associated with drying and storage conditions on the physicochemical properties quality using mathematical modeling and multivariate analysis.

## Material and methods

### Material

Soybean (*Glycine max* L.) of the cultivar BRS 7570 IPRO with an average cycle of 109 days was cultivated at a density of 360 to 380 thousand plants per hectare, in a high fertility soil, reaching a productivity of 4920 kg per hectare. Soybeans were harvested with 23% (d.b.) and 18% (d.b.) moisture content.

### Drying conditions

**Figure 1:**
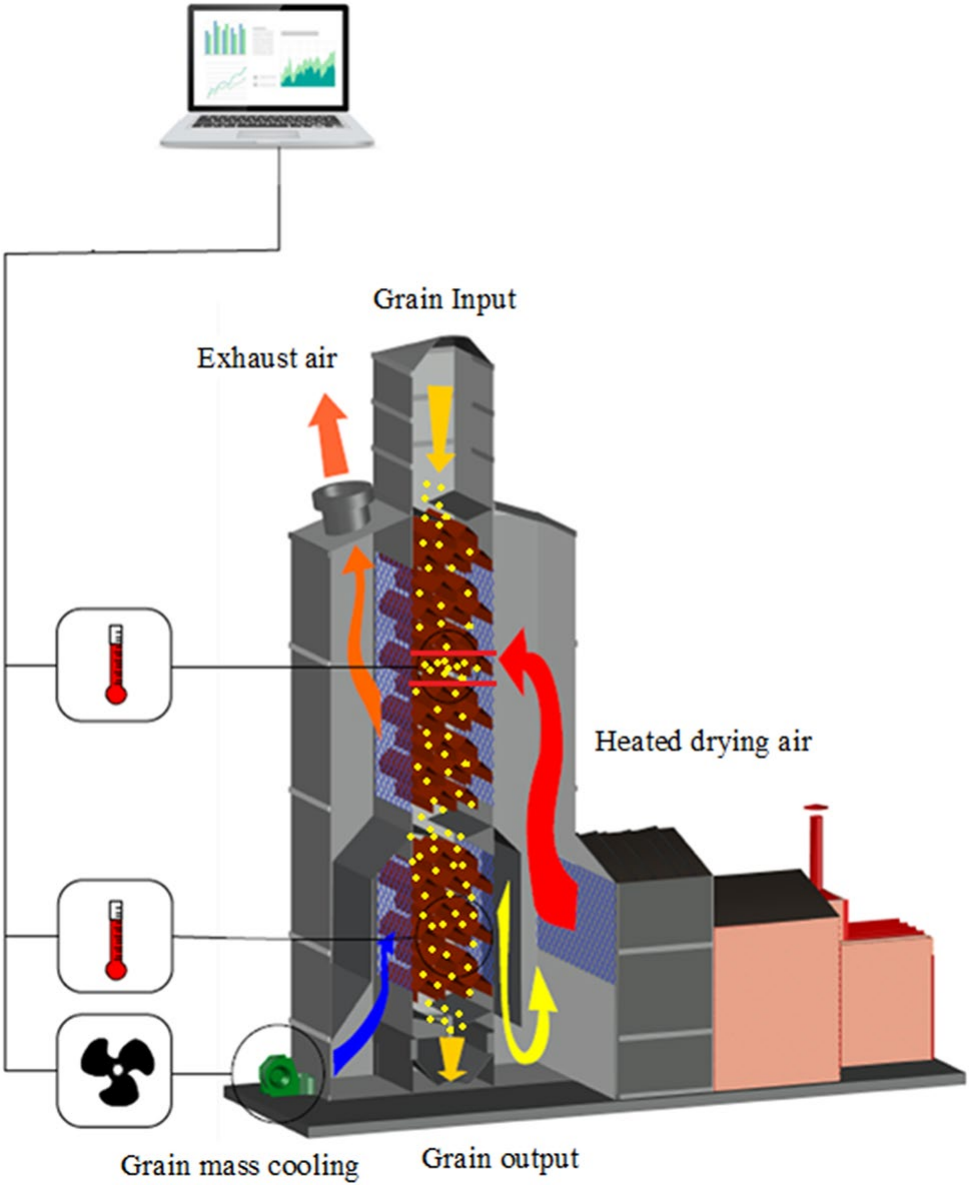
Schema of the dryer system (Software SolidWorks^@^, student version, https://www.solidworks.com/pt-br/product/students).

The soybean was subjected to drying in a continuous dryer (Fig. 1), commercial convectional model dryer-KW-Khronos, capacity 60 t h^−1^ (Kepler Weber, Panambi, Brazil), at 80, 100, and 120 °C. We consider thin layer drying due to the high airflow (238 m^3^/h) that occupies a large part of the drying chamber and crosses a thin layer of grains in downward movement. The dryer has a specific point in the drying chamber for the passage of heated air, where measurements and sampling of the grains were carried out.

Three tests were performed for each initial moisture content grain harvested (18 and 23%) and drying air temperature (80, 100, and 120 °C) for three repetitions. During drying, soybean samples were collected at 15 min intervals to determine the moisture content and volumetric shrinkage. In total was collected 102 samples of 2 kg were at the exit of the drying chamber on the bucket elevator belt. Drying was carried out until the grains reached moisture contents of 11% (d.b.). At the end of the drying, a sample of each repetition (a total of 18 samples) was collected to determine the physicochemical grain quality.

The moisture contents were measured by the indirect method of electrical capacitance using the G650i model equipment (Gehaka, São Paulo, Brazil) calibrated by drying oven method TE-394/2-MP model (Tecnal, Piracicaba, SP, Brazil), with convective heated air at 105 ± 1 °C for 24 h and forced ventilation with air. Then, the sample was sent to a desiccator with silica for cooling, for 5 min. The moisture content was calculated by the initial and final difference of the sample weight using a digital balance, model B13200H (Shimadzu, Kyoto, Japan), in three replications^25^. We also measured the temperature and relative humidity of the ambient. The temperature and relative humidity were checked with studio monitors with the aid of a psychrometer, model PY-5080 (Instrufiber, São Paulo, Brazil).

The volume (*V*_*g*_) of the fifty grains was determined at each sampling performed during the drying process with the aid of a caliper, according to the expression (1)^26^. The unitary volumetric shrinkage (*Ψg*) during the drying of the product was determined by the ratio between the final and initial volumes of the grain for each moisture content.

1 $$ {Vg = \frac{\pi abc}{{6}}} $$

where *a:* major axis of the grain (mm), *b*: mean axis of the seed (mm), *c*: minor axis of the seed (mm).

The experimental unit shrinkage, expressed by the following mathematical models have been adjusted^26,27^:

**Table:** 

Models references	Models	
Bala and Woods	$\psi_{g} = a\left\{ {1 - \exp \left[ {b(\overline{X} - X_{0} )} \right]} \right\}$	(2)
Lang and Sokhansanj	$\psi_{g} = a + \beta_{1} (\overline{X} - X_{0} )$	(3)
Rahman	$\psi_{g} = a + \beta_{2} (\overline{X} - X_{0} )$	(4)
Corrêa	$\psi_{g} = 1/\left[ {a + b\exp (\overline{X} )} \right]$	(5)
Line	$\psi_{g} = a + b\overline{X}$	(6)
Exponential	$\psi_{g} = a\exp (b\overline{X} )$	(7)

where *Ψ*_*g*_: unit volume shrinkage (d.b.), ${\overline{X} }$: moisture content of the product (d.b.), *X*_*0*_: initial moisture content of the product (d.b.), *ß*_*1*_: *a* + *b*(*UR*) + *c*(*T*), *a, b* : parameters that depend on the product, *T*: air temperature (°C), *ß*_*2*_ : volumetric coefficient, dimensionless contraction.

The drying curves were fitted to the experimental data using thirteen different semi-empirical and empirical equations^4,10,11,19,20,28–30^, discriminated below:

**Table:** 

Models	Models references	
${MR = {\text{exp}}\left( { - kt} \right)}$	Newton	(8)
${MR = {\text{exp}}\left( { - kt^{n} } \right)}$	Page	(9)
${MR = {\text{exp}}\left( { - (kt)^{n} } \right)}$	Page Modified	(10)
${MR = a{\text{exp}}\left( { - kt} \right)}$	Henderson & Pabis	(11)
${MR = a{\text{exp}}\left( { - kt} \right) + c}$	Logarithmic	(12)
${MR = a{\text{exp}}\left( { - k_{o} t} \right) + b{\text{exp}}\left( { - k_{{1}} t} \right)}$	Two Terms	(13)
${MR = a{\text{exp}}\left( { - \kappa \tau } \right) + \left( {{1} - a} \right){\text{ exp}}\left( { - kat} \right)}$	Two Exponential Terms	(14)
${MR = 1 + at + bt^{2} }$	Wang & Singh	(15)
${MR = a{\text{exp}}\left( { - kt} \right) + b{\text{exp}}( - k{}_{{0}}t) + c{\text{exp}}( - k_{{1}} t)}$	Henderson & Pabis Modified	(16)
${MR = a{\text{exp}}\left( { - kt^{n} } \right) + bt}$	Midilli	(17)
${MR = a{\text{exp}}\left( { - kt} \right) + ({1} - a){\text{exp}}( - kbt)}$	Diffusion approximation	(18)

where *MR*: moisture ratio (dimensionless), *t* : drying time (h), *k, k*_*o*_*, k*_*1*_ : drying constant (h^−1^), *a, b, c, n* : model coefficients.

For determining the ratios of moisture during drying under different conditions, the following expression was used (Eq. 19)^4,10,19,28^:

19 $$ {MR = \frac{{\overline{X} - X_{e} }}{{X_{{0}} - X_{e} }}} $$

where *X*_*e*_ : equilibrium moisture content of the product (d.b.)

In thin-layer drying of agricultural products, analysis of the dehydration process that takes place in the falling rate period is calculated using a simple diffusion model based on Fick’s second law. Evaluation of the moisture diffusion mechanism in spherical bodies can be represented by the following Eq. (20)^27,28^:

20 $$ {\frac{\partial X}{{\partial t}} = \frac{D}{{r^{2} }}\left[ {\frac{\partial }{\partial r}\left( {r^{2} \frac{\partial X}{{\partial r}}} \right)} \right]} $$

where *X:* moisture content (kg_water_/kg_DS_), *t:* time (s), *D*: diffusivity (m^2^ s^−1^), *r*: radius coordinate (m).

The method of slopes was used for the estimation of effective moisture diffusivity of soybean kernels at corresponding moisture content under different drying conditions. The uniform moisture content was assumed as the initial condition (Eq. 21). Due to the geometry, the asymmetry boundary condition was defined (Eq. 22). Finally, the second boundary condition was the neglect of external resistance (Eq. 23)^27,28^:

21 $$ {X(r{,0}) = X_{0} } $$

22 $$ {\frac{\partial X}{{\partial r}}({0},t) = {0}} $$

23 $$ {X(R,t) = X_{e} } $$

A sphere with initial moisture content, which is subjected to the drying process in the open air, under constant conditions, can be described by Fick's theory, having the following analytical solution (Eq. 24)^29,30^:

24 $$ {MR = \frac{{\overline{X} - X_{e} }}{{X_{{0}} - X_{e} }} = \frac{{6}}{{\pi^{2} }}\sum\limits_{{n = {1}}}^{\infty } {\frac{{1}}{{n^{2} }}} {\text{exp}}\left( {\frac{{ - Dn^{2} \pi^{2} t}}{{R^{{2}} }}} \right)} $$

where *R*: sphere radius (m).

It is usual to consider the value of the diffusion coefficient constant or linearly. This relationship has been expressed by the Arrhenius model (Eq. 25)^20^:

25 $$ {D = A{\text{exp}}\left( { - \frac{E}{RT}} \right)} $$

where *A*: constant (m^2^ s^−1^), *E*: activation energy (kJ kmol^−1^), *R*: universal gas constant (8314 kJ kmol^−1^ K^−1^), *T*: absolute temperature (K).

### Storage conditions

Soybeans harvested at different moisture content (18 and 23%) and dried at different temperatures (80, 100, and 120 °C) were stored in paper and plastic raffia-polyethylene bags at 15, 23, and 30 °C in climatic chambers for 0, 4, and 8 months. Three repetitions per treatment were performed. A total of 432 soybean samples were collected and submitted to physicochemical quality assessments.

### Physicochemical quality of soybeans

The moisture content, oil content, acid index, and crude protein content (% d.b.) were determined according to AOAC^25^. The electrical conductivity test was conducted in soybean, according to Vieira & Krzyzanowski^31^.

### Statistical analysis

To adjust the mathematical models of analysis of soybean drying, nonlinear regression was performed, through the Quasi-Newton method, using the computer program Statistica 7.0^®^. To check the degree of fit of each model, the significance of the regression coefficient by t-test was considered, adopting the 1 and 5% level of probability, the magnitude of the coefficient of determination (R^2^), the mean relative error values (P), the average estimated error (SE), and verified the behavior of the distribution of residuals. The relative average error and the average error estimated for each model were calculated according to the following expressions, respectively:

26 $$ P = \frac{100}{n}\sum {\frac{{\left| {Y - \hat{Y}} \right|}}{Y}} $$

27 $$ {SE = \sqrt {\frac{{\sum {\left( {Y - \hat{Y}} \right)^{{2}} } }}{GLR}} } $$

where *Y* : experimentally observed value, ${\hat{Y}}$: value calculated by the model, *n*: number of experimental observations, *GLR:* degrees of freedom of the model.

The data for physicochemical quality were analyzed by analysis of variance, Tukey's test at 1 and 5% probabilities, and linear regression. After standardizing the variables, the data were submitted to cluster analysis. For this, we use Euclidean distance and Ward's hierarchical method. After defining the groups, we constructed a graph containing the dispersion of the values of the variables and their respective Pearson correlations for each group. These analyzes were performed with the "ggfortify" and "GGally" packages from software R (Table S1).

### Ethics declarations

The experimental research and field studies on plants and plant material were comply with local and national regulations. The study complied with institutional, national, and international guidelines and legislation.

## Results and discussion

### Drying kinetics and quality of soybeans on the drying

**Figure 2:**
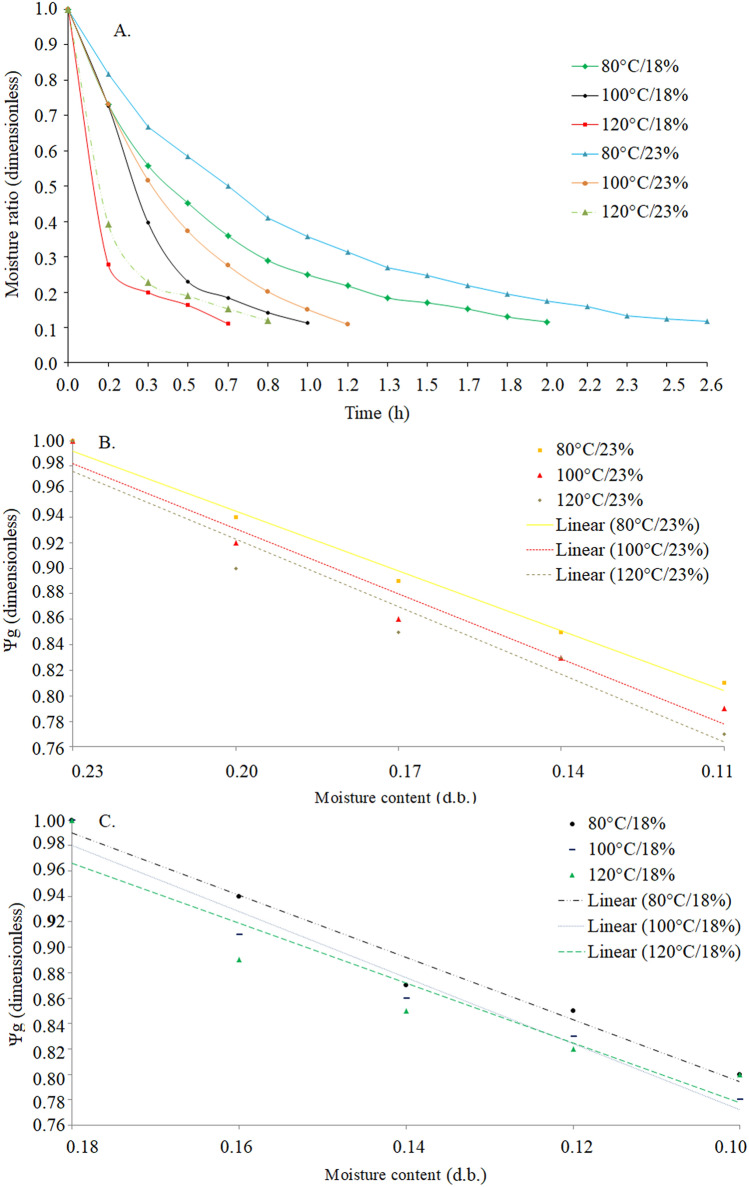
Moisture content adjusted by the Wang & Singh model (**A**), volumetric shrinkage of soybeans in the drying using the model of Rahman, at initial moisture content of 23% (d.b.) (**B**) and 18% (d.b.) (**C**).

In the results obtained (Fig. 2A), the drying curves at different temperatures describe a logical behavior and values. It was observed that the increase in the drying air temperature to lower the initial moisture content of the soybean reduced the drying time. However, at the end of the process, the grains reached the same moisture ratio. Soybeans with initial moisture contents of 23% (d.b.) and drying at 80 °C completed the drying process in a higher time of 2.6 h, while soybean with initial moisture contents of 18% (d.b.) subjected at 120 °C took 0.7 h to complete the process. The other conditions evaluated varied the drying time from 0.8 to 2.0 h. During the drying period, the ambient air temperature varied between 22 and 26 °C and the relative humidity between 50 and 65%. Regardless of the initial moisture contents, in the final third of drying with an air temperature above 100 °C there was an increase in the temperature of the grain mass to 45 °C, while in the drying at 80 °C and from the middle of the process, the soybean remained with a mass temperature between 36 and 38 °C.

**Figure 3:**
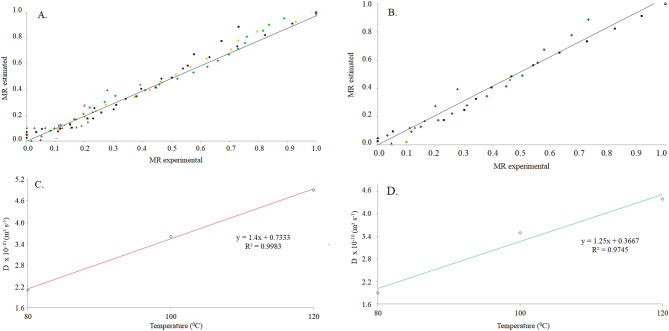
Ratio of experimental values and estimated by the Wang & Singh model at initial moisture content of 23% (d.b.) (**A**), and at initial moisture content of 18% (d.b.) (**B**), effective diffusion coefficient (m^2^ s^−1^) for 23% (d.b.) of the initial moisture content in the grains (**C**), effective diffusion coefficient (m^2^ s^−1^) for 18% (d.b.) of the initial moisture content in the grains (**D**).

The coefficients of the adjusted models analyzed during the drying of soybean are shown in Tables 1 and 3. The coefficients of determination R^2^ indicated a satisfactory representation of the phenomenon under study (Tables 2 and 4). Among all tested models, Wang and Singh's model showed the lower values of the mean relative error (P), average estimated error (SE) (Table 3), and distribution of residues for the temperature of the air drying 80, 100, and 120 °C (Tables 2 and 4). Thus, the experimental drying results fit satisfactorily with the estimated data (Fig. 3A, B). It was observed that soybean with an initial moisture content of 23% (d.b.) had a better fit (Fig. 3A) for the estimated and experimental moisture ratio values in drying. This may have occurred due to the longer drying time and homogeneity, especially at temperatures of 80 and 100 °C.

**Table 1:** Parameters obtained from models fitted to the data for drying of soybean grains for 23% (d.b.) of initial moisture content.

**Mathematical models**	T (°C)	*k*					
Newton	80	0.463600					
	100	0.662870					
	120	7.602332					

	T (°C)	*k*	*n*				
Page	80	0.293530	1.617070				
	100	0.388050	1.302200				
	120	0.499964	0.000125				

	T (°C)	*k*	*n*				
Page modified	80	0.468590	1.617070				
	100	0.665160	1.302200				
	120	0.046526	0.000017				

	T (°C)	*a*	*k*				
Henderson & Pabis	80	1.064980	0.488950				
	100	1.047710	0.699440				
	120	0.989430	7.531181				

	T (°C)	*a*	*k*	*c*			
Logarithmic	80	11.16890	0.024820	− 10.189600			
	100	2.704230	0.157990	− 1.732700			
	120	0.930360	9.952775	0.067291			

	T (°C)	*a*	*k*_*0*_	*b*	*k*_*1*_		
Two terms	80	0.532496	0.498886	0.532496	0.498860		
	100	0.523857	0.699442	0.523557	0.699442		
	120	0.494720	7.531181	0.494720	7.531181		

	T (°C)	*a*	*k*				
Two exponential terms	80	1.938070	0.726790				
	100	1.796970	0.955320				
	120	0.317576	17.99533				

	T (°C)	*a*	*b*				
Wang & Singh	80	− 0.284900	0.002680				
	100	− 0.462100	0.039562				
	120	− 4.089220	4.080789				

	T (°C)	*a*	*k*	*b*	*k*_*0*_	*c*	*k*_*1*_
Henderson & Pabis modified	80	0.354997	0.498886	0.354997	0.198886	0.354997	0.498886
	100	0.349238	0.699442	0.349238	0.699442	0.349238	0.699442
	120	0.329811	7.531181	0.329811	7.531181	0.329811	7.531181

	T (°C)	*a*	*k*	*n*	*b*		
Midilli	80	0.990558	0.023506	0.000019	− 0.264911		
	100	1.003716	0.175032	0.519332	− 0.268476		
	120	0.771639	0.626056	0.000218	− 0.684975		

	T (°C)	*a*	*k*	*b*			
Diffusion approximation	80	0.541710	0.464301	1.000000			
	100	0.528730	0.662866	0.999900			
	120	0.409782	9.255399	55.000000

**Table 2:** Coefficient of determination (R^2^), mean relative error (P), estimated values ​​of average error (SE) drying of soybean grains due to different temperatures for 23% (d.b.) of initial moisture content.

Mathematical models	80 °C	100 °C	120 °C
	**R**^**2**^** (%)**		
Newton	95.17	97.70	98.67
Page	98.12	98.76	94.15
Page modified	98.12	98.76	76.57
Henderson & Pabis	95.55	97.92	98.68
Logarithmic	99.37	99.68	99.27
Two terms	95.57	97.92	98.68
Two exponential terms	97.50	98.69	99.14
Wang & Singh	99.32	99.59	99.04
Henderson & Pabis modified	95.57	97.92	98.68
Midilli	99.36	97.78	95.14
Diffusion approximation	95.19	97.70	99.73
	**SE**		
Newton	0.0890	0.0626	0.06488
Page	0.0594	0.0477	0.56505
Page modified	0.0594	0.0477	0.29748
Henderson & Pabis	0.0911	0.0603	0.07467
Logarithmic	0.0347	0.0603	0.05569
Two terms	0.0948	0.0648	0.12933
Two exponential terms	0.0689	0.0603	0.06035
Wang & Singh	0.0321	0.0204	0.01717
Henderson & Pabis modified	0.1002	0.0705	0.12933
Midilli	0.0345	0.0214	0.24655
Diffusion approximation	0.0974	0.0670	0.04121
	**P (%)**		
Newton	6.85	0.24	2.79
Page	5.98	7.16	5.60
Page modified	5.98	6.16	8.41
Henderson & Pabis	5.85	4.82	4.11
Logarithmic	1.74	4.82	1.47
Two terms	6.55	4.82	4.11
Two exponential terms	4.97	4.82	6.11
Wang & Singh	3.01	3.45	2.52
Henderson & Pabis modified	6.55	4.82	4.11
Midilli	1.34	0.27	4.64
Diffusion approximation	7.39	6.24	7.35
	**Distribution residue**		
Newton	T	T	T
Page	T	T	A
Page modified	T	T	A
Henderson & Pabis	T	T	A
Logarithmic	A	A	A
Two terms	T	T	A
Two exponential terms	T	A	A
Wang & Singh	A	A	A
Henderson & Pabis modified	T	T	A
Midilli	A	A	A
Diffusion approximation	T	T	A

**Table 3:** Parameters obtained from models fitted to the data for drying of soybean grains for 18% (d.b.) of initial moisture content.

**Mathematical models**	T (°C)	*k*					
Newton	80	0.971060					
	100	5.889470					
	120	5.786360					

	T (°C)	*k*	*n*				
Page	80	0.974770	0.976759				
	100	0.620970	0.000086				
	120	0.672511	0.000004				

	T (°C)	*k*	*n*				
Page modified	80	0.974180	0.976759				
	100	0.044058	0.000038				
	120	0.044463	0.000079				

	T (°C)	*a*	*k*				
Henderson & Pabis	80	0.955030	0.924030				
	100	0.970820	5.691402				
	120	1.001650	5.793869				

	T (°C)	*a*	*K*	*c*			
Logarithmic	80	1.430271	0.405995	− 0.534078			
	100	0.912190	8.981745	0.081508			
	120	1.071784	4.830920	− 0.076094			

	T (°C)	*a*	*k*_*0*_	*B*	*k*_*1*_		
Two terms	80	0.477517	0.924032	0.477517	0.924032		
	100	0.485410	5.691402	0.485410	5.691402		
	120	0.500825	5.753869	0.500825	5.793869		

	T (°C)	*a*	*k*				
Two exponential terms	80	1.390710	1.090440				
	100	0.291287	14.96863				
	120	1.481680	6.748937				

	T (°C)	*a*	*b*				
Wang & Singh	80	− 0.718800	0.132126				
	100	− 2.820600	1.925448				
	120	− 4.050820	4.181176				

	T (°C)	*a*	*k*	*b*	*k*_*0*_	*c*	*k*_*1*_
Henderson & Pabis modified	80	0.318345	0.924032	0.318345	0.924032	0.318345	0.924032
	100	0.323610	5.691402	0.323610	5.691402	0.323610	5.691402
	120	0.333883	5.793869	0.333883	5.793869	0.333883	5.793869

	T (°C)	*a*	*k*	*n*	*b*		
Midilli	80	0.945430	0.190295	0.000044	− 0.34510		
	100	0.785988	0.511196	0.000012	0.610212		
	120	1.000000	1.329180	0.373421	0.716832		

	T (°C)	*a*	*k*	*b*			
Diffusion approximation	80	0.569273	0.97106	1.00000			
	100	0.414057	2.25745	1.03247			
	120	9.285317	7.67864	1.03917

**Table 4:** Coefficient of determination (R^2^), mean relative error (P), estimated values ​​of average error (SE) drying of soybean grains due to different temperatures for 18% (d.b.) of initial moisture content.

Mathematical models	80 °C	100 °C	120 °C
	**R**^**2**^** (%)**		
Newton	97.57	97.25	99.64
Page	97.58	97.63	57.08
Page modified	97.58	67.04	83.51
Henderson & Pabis	97.76	97.31	99.64
Logarithmic	98.88	98.64	99.79
Two terms	97.76	97.31	99.64
Two exponential terms	97.61	98.26	99.67
Wang & Singh	97.47	88.59	99.30
Henderson & Pabis modified	97.76	97.31	99.64
Midilli	99.75	93.40	99.99
Diffusion approximation	97.57	99.75	99.67
	**SE**		
Newton	0.0618	0.0792	0.0366
Page	0.0640	0.4547	0.4378
Page modified	0.0640	0.2769	0.7287
Henderson & Pabis	0.0615	0.0859	0.8021
Logarithmic	0.0437	0.0612	0.8138
Two terms	0.0669	0.1109	0.5355
Two exponential terms	0.0636	0.0693	0.8064
Wang & Singh	0.0267	0.0170	0.0260
Henderson & Pabis modified	0.0740	0.1920	1.1344
Midilli	0.0224	0.1721	1.1528
Diffusion approximation	0.0668	0.0291	1.1411
	**P (%)**		
Newton	2.37	7.14	7.44
Page	2.17	9.65	6.42
Page modified	2.17	2.18	6.55
Henderson & Pabis	8.88	4.67	6.16
Logarithmic	5.13	4.37	5.55
Two terms	8.88	4.67	9.45
Two exponential terms	8.32	6.21	9.36
Wang & Singh	2.20	2.37	2.30
Henderson & Pabis modified	8.88	4.67	9.16
Midilli	2.78	9.18	9.65
Diffusion approximation	6.00	9.67	9.99
	**Distribution residue**		
Newton	T	A	A
Page	T	A	A
Page modified	T	A	A
Henderson & Pabis	T	T	A
Logarithmic	A	A	A
Two terms	T	T	A
Two exponential terms	T	T	A
Wang & Singh	A	A	A
Henderson & Pabis modified	T	T	A
Midilli	A	A	A
Diffusion approximation	T	A	A

These findings are consistent with that published recently^32–34^. The moisture remotion occurs fast in the first half of the process, afterwards, it is slower since the diffusion of the water in the grain's inner geometry is more difficult to happen. Even though the process parameters require an air temperature of 100 °C to obtain a faster drying, the literature recommends that the temperature should be lower, so as not to damage the structure of soybeans and accelerate degradation. It was found that the effects of initial moisture content and temperature on drying time were proportional, which means that both factors influenced the soybean quality.

The effective diffusion coefficient increased significantly and with a uniform variation, with the increase of the drying air temperature (Fig. 3C, D) for a linear adjustment^27,30,35,36^, being the higher values of diffusion obtained in the drying with initial moisture contents of 23% (d.b.). Thus, the diffusivity results reflected on soybean volumetric shrinkage, being that it was 23.20% for moisture content of 0.18 to 0.11 (d.b.) and 21.1% for moisture content of 0.23 to 0.11 (d.b.) (Fig. 2B, C). In this study, the Rahman model was the best set of data obtained volumetric shrinkage of soybeans, with a less pronounced trend of distribution of residuals (random distribution) (Tables 5 and 6). These models had a higher coefficient of determination and lower estimates and average errors relative. Thus, the Rahman model was recommended to predict the phenomenon of shrinkage of the soybean.

**Table 5:** Parameters estimated, coefficient of determination (R^2^), estimated average (SE) ​​and relative error (P) and distribution of residues of the mathematical models used to describe the shrinkage of soybeans grains to different drying air temperatures and an initial moisture content of the grains of 23% (d.b.).

Mathematical models	Estimation of parameters	R^2^	SE (decimal)	P (%)	Distribution of residuals
**80 °C**					
Bala and Woods	*a* = 0.94693 *b* = − 17.9467	72.21	0.039817	2.275428	A
Lang and Sokhansanj	*a* = 2.31625	82.12	0.035181	2.136110	A
Rahman	*b* = 1.17238	93.22	0.018143	0.985810	A
	*a* = 0.27142				
Corrêa	*a* = 0.70713	90.60	0.024360	1.000093	A
	*b* = 1.05963				
Line	*a* = 2.51031 *b* = − 1.16293	92.99	0.021167	1.044430	A
Exponential	*a* = 0.72095 *b* = 1.21226	91.49	0.023226	2.025056	A
**100 °C**					
Bala and Woods	*a* = 0.99175 *b* = − 16.9372	98.07	0.011486	1.238779	A
Lang and Sokhansanj	*a* = 2.37193	81.48	0.012131	2.617105	A
	*b* = 1.21561				
Rahman	*a* = 0.27138	99.61	0.009125	0.987234	A
Corrêa	*a* = 0.72227	98.50	0.010429	1.013273	A
	*b* = 1.14054				
Line	*a* = 2.41417 *b* = − 1.11077	97.37	0.013501	1.036152	A
Exponential	*a* = 0.74136 *b* = 1.23357	98.15	0.011486	1.238779	A
**120 °C**					
Bala and Woods	*a* = 1.10554 *b* = − 16.0361	98.22	0.0148753	2.1352353	A
Lang and Sokhansanj	*a* = 3.35812	88.50	0.0426990	1.5287221	A
	*b* = 1.24877				
Rahman	*a* = 0.341239	99.67	0.0141121	0.9146112	A
Corrêa	*a* = 0.52231	99.23	0.0141216	3.4515100	A
	*b* = 1.36381				
Line	*a* = 2.14782 *b* = − 1.12214	96.88	0.0194607	1.7472132	A
Exponential	*a* = 0.81248 *b* = 1.24151	94.19	0.0160931	3.2332221	A

**Table 6:** Parameters estimated, coefficient of determination (R^2^), estimated average (SE) ​​and relative error (P) and distribution of residues of the mathematical models used to describe the shrinkage of soybeans grains to different drying air temperatures and an initial moisture content of the grains of 18% (d.b.).

Mathematical models	Estimation of parameters	R^2^	SE (decimal)	P (%)	Distribution of residuals
**80 °C**					
Bala and Woods	*a* = 0.96144 *b* = − 18.32210	95.71	0.035730	1.195070	A
Lang & Sokhansanj	*a* = 2.14567	98.21	0.046570	2.345619	A
Rahman	*b* = 1.34560	99.23	0.018233	1.023451	A
	*a* = 0.23450				
Corrêa	*a* = 0.68100	95.85	0.021048	1.100000	A
	*b* = 1.40000				
Line	a = 2.97552 *b* = − 1.59233	97.67	0.026304	1.042430	A
Exponential	*a* = 0.69567 *b* = 1.60571	96.55	0.027392	2.021655	A
**100 °C**					
Bala and Woods	*a* = 1.055487 *b* = − 13.4491	94.46	0.0249491	1.3962264	A
Lang & Sokhansanj	*a* = 2.21572	90.23	0.0317891	1.4527809	A
	*b* = 1.41018				
Rahman	*a* = 0.31017	98.45	0.0061234	1.0345167	A
Corrêa	*a* = 0.586533	97.56	0.0128582	1.1006289	A
	*b* = 2.12000				
Line	*a* = 3.769244 *b* = − 2.28705	99.13	0.0051166	1.1773585	A
Exponential	*a* = 0.631492 *b* = 2.380158	97.87	0.0113594	2.0207547	A
**120 °C**					
Bala and Woods	*a* = 1.023189 *b* = − 13.1101	95.31	0.0123410	2.2981331	A
Lang & Sokhansanj	*a* = 2.12312	92.34	0.0123145	3.1901231	A
	*b* = 1.32191				
Rahman	*a* = 0.22141	99.48	0.0341678	2.0245178	A
Corrêa	*a* = 0.42145	98.21	0.0412891	4.2314561	A
	*b* = 2.21344				
Line	*a* = 3.51234 *b* = − 2.12341	96.45	0.0532156	3.5414579	A
Exponential	*a* = 0.342141 *b* = 2.10231	99.41	0.0651294	3.1234526	A

The results obtained in this study confirm that drying has immediate effects on soybean quality (Tables 7 and 8). Drying at air temperatures above 100 °C negatively affects the physicochemical quality, mainly in soybeans harvested with 18% moisture (Tables 7 and 8 time zero). Similar results were observed by Mourad et al^37^ and Wang et al^22^ when evaluating the effect of temperature on the grain drying. It is observed that the grain cell has been compromised grain structure along with the different drying air temperatures, the higher the amount of ions leached at the drying temperature of 120 °C. The damage to the cell walls of grains causing high values of electrical conductivity affects the oil content and acidity. The increase in electrical conductivity may be implicated in the major damage caused by the drying air temperature on the soybean cellular structure during drying, causing them to lose physiological and nutritional quality^38,39^.

**Table 7:** Quality of soybeans harvest at 23% (d.b.) moisture content subjected to drying at 80, 100 and 120 °C, stored in different environments and packaging for eight months.

Analysis	Times (months)	Storage conditions					
		15 °C		23 °C		30 °C	
		P	PL	P	PL	P	PL
		**Drying air temperature at 80 °C**					
Moisture content (% d.b.)	0	10.31 Ba	10.31 Ba	10.31 Aa	10.31 Aa	10.31 Aa	10.31 Ba
	4	10.24 Ba	10.22 Ba	10.27 Aa	10.20 Aa	10.20 Aa	10.28 Ba
	8	11.20 Ab	12.00 Aa	9.90 Bd	10.50 Ac	9.20 Bd	11.00 Ab
Conductivity electrical (µS cm^−1^ g^−1^)	0	191 Ca	191 Ca	191 Ca	191 Ca	191 Ca	191 Ca
	4	205 Bc	196 Bd	220 Bb	197 Bd	241 Ba	209 Bc
	8	235 Ad	210 Ae	320 Ab	210 Ae	391 Aa	254 Ac
Oil content (%)	0	25.89 Aa	25.89 Aa	25.89 Aa	25.89 Aa	25.89 Aa	25.89 Aa
	4	23.10 Ba	23.70 Ba	22.00 Bb	23.50 Ba	22.12 Bb	22.45 Bb
	8	21.29 Cb	22.27 Ca	20.29 Cc	22.27 Ca	19.89 Cd	20.50 Cc
Index of acidity (mL)	0	5.54 Aa	5.54 Aa	5.54 Ba	5.54 Aa	5.54 Ba	5.54 Ba
	4	5.75 Aa	5.58 Aa	5.78 Ba	5.60 Aa	5.62 Ba	5.61 Ba
	8	5.80 Ac	5.60 Ac	6.02 Ab	5.67 Ac	7.71 Aa	6.69 Ab
Crude protein (%)	0	35.69 Aa	35.69 Aa	35.69 Aa	35.69 Aa	35.69 Aa	35.69 Aa
	4	31.15 Bd	34.36 Ba	32.15 Bc	33.54 Bb	28.24 Be	30.45 Bf
	8	30.34 Cc	33.45 Ca	28.35 Cd	31.23 Cb	25.34 Cf	27.74 Ce
		**Drying air temperature at 100 °C**					
Moisture content (% d.b.)	0	10.23 Ba	10.23 Ba	10.23 Aa	10.23 Aa	10.23 Aa	10.23 Ba
	4	10.12 Ba	10.13 Ba	10.41 Aa	10.26 Aa	10.39 Aa	10.20 Ba
	8	11.11 Ab	12.09 Aa	10.10 Bd	10.59 Ac	9.32 Bd	11.09 Ab
Conductivity electrical (µS cm^−1^ g^−1^)	0	200 Ca	200 Ca	200 Ca	200 Ca	200 Ca	200 Ca
	4	215 Bc	199 Bd	225 Bb	199 Bd	262 Ba	218 Bc
	8	243 Ad	217 Ae	329 Ab	214 Ae	399 Aa	264 Ac
Oil content (%)	0	24.19 Aa	24.19 Aa	24.19 Aa	24.19 Aa	24.19 Aa	24.19 Aa
	4	22.10 Ba	22.51 Ba	21.14 Bb	22.42 Ba	21.19 Bb	21.85 Bb
	8	20.13 Cb	21.16 Ca	19.67 Cc	21.36 Ca	18.65 Cd	19.66 Cc
Index of acidity (mL)	0	5.75 Aa	5.75 Aa	5.75 Ba	5.75 Aa	5.75 Ba	5.75 Ba
	4	5.85 Aa	5.69 Aa	5.89 Ba	5.79 Aa	5.76 Ba	5.86 Ba
	8	6.10 Ac	5.84 Ac	6.12 Ab	5.92 Ac	7.98 Aa	6.71 Ab
Crude protein (%)	0	34.39 Aa	34.39 Aa	34.39 Aa	34.39 Aa	34.39 Aa	34.39 Aa
	4	30.43 Bd	33.54 Ba	31.31 Bc	32.44 Bb	27.42 Be	29.47 Bf
	8	29.36 Cc	32.55 Ca	27.47 Cd	30.13 Cb	24.14 Cf	26.36 Ce
		**Drying air temperature at 120 °C**					
Moisture content (% d.b.)	0	10.40 Ba	10.40 Ba	10.40 Aa	10.40 Aa	10.40 Aa	10.40 Ba
	4	10.56 Ba	10.62 Ba	10.60 Aa	10.55 Aa	10.51 Aa	10.62 Ba
	8	11.28 Ab	12.25 Aa	10.06 Bd	10.40 Ac	9.21 Bd	11.10 Ab
Conductivity electrical (µS cm^−1^ g^−1^)	0	208 Ca	208 Ca	208 Ca	208 Ca	208 Ca	208 Ca
	4	224 Bc	210 Bd	244 Bb	206 Bd	279 Ba	245 Bc
	8	265 Ad	229 Ae	337 Ab	222 Ae	414 Aa	296 Ac
Oil content (%)	0	23.34 Aa	23.34 Aa	23.34 Aa	23.34 Aa	23.34 Aa	23.34 Aa
	4	21.11 Ba	21.76 Ba	20.54 Bb	21.29 Ba	20.57 Bb	20.72 Bb
	8	18.54 Cb	20.18 Ca	18.75 Cc	20.61 Ca	17.45 Cd	18.58 Cc
Index of acidity (mL)	0	6.15 Aa	6.15Aa	6.15 Ba	6.15Aa	6.15 Ba	6.15 Ba
	4	6.45 Aa	6.79 Aa	6.80 Ba	6.83 Aa	6.66 Ba	6.76 Ba
	8	6.60 Ac	6.93 Ac	6.99 Ab	6.92 Ac	8.18 Aa	7.51 Ab
Crude protein (%)	0	33.56 Aa	33.56 Aa	33.56 Aa	33.56 Aa	33.56 Aa	33.56 Aa
	4	31.13 Bd	33.54 Ba	31.31 Bc	32.44 Bb	26.57 Be	28.33 Bf
	8	28.55 Cc	31.76 Ca	26.41 Cd	29.10 Cb	23.11 Cf	25.61 Ce
		**Mixed grains (80/100/120 °C)**					
Moisture content (% d.b.)	0	10.11 Ba	10.11 Ba	10.11 Aa	10.11 Aa	10.11 Aa	10.11 Ba
	4	10.19 Ba	10.15 Ba	10.17 Aa	10.22 Aa	10.10 Aa	10.29 Ba
	8	11.09 Ab	11.80 Aa	9.85 Bd	10.42 Ac	9.10 Bd	10.87 Ab
Conductivity electrical (µS cm^−1^ g^−1^)	0	205 Ca	205 Ca	205 Ca	205 Ca	205 Ca	205 Ca
	4	213 Bc	202 Bd	222 Bb	201 Bd	258 Ba	221 Bc
	8	239 Ad	219 Ae	322 Ab	217 Ae	402 Aa	260 Ac
Oil content (%)	0	24.10 Aa	24.10Aa	24.10Aa	24.10Aa	24.10Aa	24.10Aa
	4	22.14 Ba	22.43 Ba	21.08 Bb	22.41 Ba	21.21 Bb	21.15 Bb
	8	20.35 Cb	21.40 Ca	19.19 Cc	21.45 Ca	18.76 Cd	19.30 Cc
Index of acidity (mL)	0	5.62 Aa	5.62 Aa	5.62 Ba	5.62 Aa	5.62 Ba	5.62 Ba
	4	5.78 Aa	5.52 Aa	5.75 Ba	5.68 Aa	5.68 Ba	5.71 Ba
	8	6.11 Ac	5.70 Ac	6.19 Ab	5.87 Ac	7.76 Aa	6.60 Ab
Crude protein (%)	0	34.68 Aa	34.68 Aa	34.68 Aa	34.68 Aa	34.68 Aa	34.68 Aa
	4	30.39 Bd	33.78 Ba	31.85 Bc	32.68 Bb	27.88 Be	29.47 Bf
	8	29.16 Cc	32.77 Ca	27.60 Cd	30.48 Cb	24.56 Cf	26.65 Ce

Means followed by the capital letter in the column for each time of storage and lower lines for each temperature of storage. do not differ at 1 and 5% probability.

PL—polyethylene plastic bag. P—paper bag.

**Table 8:** Quality of soybeans harvest at 18% (d.b.) moisture content subjected to drying at 80, 100 and 120 °C, stored in different environments and packaging for eight months.

Analysis	Times (months)	Storage conditions					
		15 °C		23 °C		30 °C	
		P	PL	P	PL	P	PL
		**Drying air temperature at 80 °C**					
Moisture content (% d.b.)	0	10.20 Ba	10.20 Ba	10.20 Ba	10.20 Ba	10.20 Aa	10.20 Ba
	4	10.56 Ba	10.13 Bb	10.41 Aa	10.10 Bb	10.12 Ab	10.19 Bb
	8	11.36 Aa	11.45 Aa	10.10 Bc	10.90 Ab	9.57 Bc	11.15 Aa
Conductivity electrical (µS cm^−1^ g^−1^)	0	199 Ca	199 Ca	199 Ca	199 Ca	199 Ca	199 Ca
	4	211 Bc	206 Bc	232 Ba	212 B	258 Ba	222 Bb
	8	244 Ac	215 A	333 Ab	221 Ad	410 Aa	276 Ac
Oil content (%)	0	24.11 Aa	24.11 Aa	24.11 Aa	24.11 Aa	24.11 Aa	24.11 Aa
	4	22.80 Ba	23.10 Ba	22.05 Bc	22.86 Ba	21.14 Bb	20.45 Bc
	8	20.75 Ca	21.04 Ca	20.00 Cb	20.90 Ca	19.00 Cc	18.79 Cd
Index of acidity (mL)	0	5.75 Ba	5.75 Aa	5.75 Ba	5.75 Ba	5.75 Ca	5.75 Ca
	4	5.90 Bc	5.85 Ac	5.85 Bc	5.80 Bc	6.15 Bb	6.68 Ba
	8	6.10 Ac	5.90 Ac	6.12 Ac	5.96 Ac	8.64 Aa	8.10 Ab
Crude protein (%)	0	35.00 Aa	35.00 Aa	35.00 Aa	35.00 Aa	35.00 Aa	35.00 Aa
	4	31.15 Bb	34.06 Ba	30.55 Bc	32.48 Bb	27.49 Bd	29.15 Bc
	8	30.34 Cb	33.15 Ca	28.10 Cc	30.13 Cb	24.89 Cd	26.80 Cc
		**Drying air temperature at 100 °C**					
Moisture content (% d.b.)	0	10.05 Ca	10.05 Ba	10.05 Ba	10.05 Ba	10.05 Aa	10.05 Ba
	4	10.41 Ba	9.98 Bb	10.26 Aa	9.95 Bb	9.97 Ab	10.04 Bb
	8	11.21 Aa	11.30 Aa	9.95 Bb	10.75 Aa	9.42 Bb	11.00 Aa
Conductivity electrical (µS cm^−1^ g^−1^)	0	219 Ca	219 Ca	219 Ca	219 Ca	219 Ca	219 Ca
	4	231 BC	226 BC	252 Bb	232 BC	278 Ba	242 BB
	8	264 Ad	235 Ad	353 Ab	241 Ad	430 Aa	296 Ac
Oil content (%)	0	23.86 Aa	23.86 Aa	23.86 Aa	23.86 Aa	23.86 Aa	23.86 Aa
	4	22.55 Ba	22.85 Ba	21.80 Bb	22.61 Ba	20.89 Bc	20.20 Bc
	8	20.50 Ca	20.79 Ca	19.75 Cb	20.65 Ca	18.75 Cc	18.54 Cc
Index of acidity (mL)	0	5.90 Ca	5.90 Ba	5.90 Ca	5.90 Ba	5.90 Ca	5.90 Ca
	4	6.05 Bb	6.00 Ab	6.00 Bb	5.95 Bb	6.30 Ba	6.83 Ba
	8	6.25 Ab	6.05 Ac	6.27 Ab	6.11 Ab	8.79 Aa	8.25 Aa
Crude protein (%)	0	35.18 Aa	35.18 Aa	35.18 Aa	35.18 Aa	35.18 Aa	35.18 Aa
	4	31.33 Bc	34.24 Ba	30.73 Bc	32.66 Bb	27.67 Be	29.33 Bd
	8	30.52 Cb	33.33 Ca	28.28 Cc	30.31 Cb	25.07 Cd	26.98 Cd
		**Drying air temperature at 120 °C**					
Moisture content (% d.b.)	0	9.92 Ca	9.92 Ba	9.92 Ba	9.92 Ba	9.92 Aa	9.92 Ba
	4	10.28 Ba	9.85 Bb	10.13 Aa	9.82 Bb	9.84 Bb	9.91 Bb
	8	11.08 Aa	11.17 Aa	9.82 Bc	10.62 Ab	9.29 Cc	10.87 Ab
Conductivity electrical (µS cm^−1^ g^−1^)	0	244 Ca	244 Ca	244 Ca	244 Ca	244 Ca	244 Ca
	4	256 Bc	251 Bc	277 Bb	257 Bc	303 Ba	267 Bb
	8	289 Ab	260 Ad	378 Ab	266 Ad	455 Aa	321 Ac
Oil content (%)	0	23.60 Aa	23.60 Aa	23.60 Aa	23.60 Aa	23.60 Aa	23.60 Aa
	4	22.29 Ba	22.59 Ba	21.54 Bb	22.35 Ba	20.63 Bc	19.94 Bd
	8	20.24 Ca	20.53 Ca	19.49 Cb	20.39 Ca	18.49 Cc	18.28 Bc
Index of acidity (mL)	0	6.03 Ba	6.03 Aa	6.03 Ba	6.03 Ba	6.03 Ca	6.03 Ca
	4	6.18 Bb	6.13 Ab	6.13 Bb	6.08 Bb	6.43 Ba	6.96 Ba
	8	6.38 Ab	6.18 Ab	6.40 Ab	6.24 Ab	8.92 Aa	8.38 Aa
Crude protein (%)	0	35.01 Aa	35.01 Aa	35.01 Aa	35.01 Aa	35.01 Aa	35.01 Aa
	4	31.16 Bc	34.07 Ba	30.56 B	32.49 Bb	27.50 Be	29.16 Bd
	8	30.35 Bb	33.16 Ca	28.11 Cc	30.14 Cb	24.90 Ce	26.81 Cd
		**Mixed grains (80/100/120 °C)**					
Moisture content (% d.b.)	0	10.17 Ca	10.17 Ba	10.17 Ba	10.17 Ba	10.17 Aa	9.92 Ba
	4	10.53 Ba	10.10 Bb	10.38 Aa	10.07 Bb	10.09 Bb	9.91 Bb
	8	11.33 Aa	11.42 Aa	10.07 Bc	10.87 Ab	9.54 Bc	10.87 Ab
Conductivity electrical (µS cm^−1^ g^−1^)	0	214 Ca	214 Ca	214 Ca	214 Ca	214 Ca	244 Ca
	4	226 Bc	221 Bc	247 Bb	227 Bc	273 Ba	267 Ba
	8	259 Ac	230 Ac	348 Ab	236 Ac	425 Aa	321 Ab
Oil content (%)	0	23.95 Aa	23.95 Aa	23.95 Aa	23.95 Aa	23.95 Aa	23.60 Aa
	4	22.64 Ba	22.94 Ba	21.89 Bc	22.70 Ba	20.98 Bd	19.94 Be
	8	20.59 Ca	20.88 Ca	19.84 Cb	20.74 Ca	18.84 Cc	18.28 Cc
Index of acidity (mL)	0	5.85 Ca	5.85 Aa	5.85 Ba	5.85 Ba	5.85 Ca	6.03 Ca
	4	6.00 Bb	5.95 Ab	5.95 Bb	5.90 Bb	6.25 Ba	6.96 Ba
	8	6.20 Ab	6.00 Ab	6.22 Ab	6.06 Ab	8.74 Aa	8.38 Aa
Crude protein (%)	0	35.26 Aa	35.26 Aa	35.26 Aa	35.26 Aa	35.26 Aa	35.01 Aa
	4	31.41 Bb	34.32 Ba	30.81 Bc	32.74 Bb	27.75 Be	29.16 Bd
	8	30.60 Cb	33.41 Ca	28.36 Cc	30.39 Cb	25.15 Cd	26.81 Cd

Means followed by the capital letter in the column for each time of storage and lower lines for each temperature of storage. do not differ at 1 and 5% probability.

PL—polyethylene plastic bag. P—paper bag.

Comparing the evaluations of volumetric shrinkage (Fig. 2A, B) and oil yield (Tables 7 and 8), it was found that a 5% reduction in the volume of the grains provided a 4.88% decrease in the oil yield extracted. The comparative results of shrinkage of grains (Fig. 2B, C), soybean oil content extracted and electrical conductivity (Tables 7 and 8), due to the effects of drying temperature and initial moisture content. According to the increase in drying temperature, a reduction in soybean oil extraction yield was observed. According to Timm et al^40^, the drying temperature from 30 to 90 °C can reduce the corn starch extraction yield by 10%. When drying was performed at 23 to 11% moisture content (d.b.) (Fig. 2A) there was a reduction of 20, 21, and 23% in the grain volume for temperatures of 80, 100, and 120 °C (Fig. 2B), respectively, while the oil content was 25.89%, 24.19%, 23.34%, respectively.

Although the diffusion process was more intense in soybeans with an initial moisture content of 23% (d.b.) compared to 18% (d.b.), mainly for the drying at 120 °C, the effects on quality in oil yield, acid index, and crude protein were better. This fact is suggested by the anticipation of soybean harvest, minimizing the effects of natural drying on the plant. Thus, harvesting with 23% (d.b.) moisture content allows the drying of the beans more slowly at a temperature around 80 °C to obtain better quality (Tables 7 and 8). Harvesting soybeans with 18% moisture content, in addition to the adverse effects of the climate that the grains were subjected to, still needs to be subjected to faster drying at a higher temperature for more efficiency in the process.

### Quality of soybeans on the storage

The early harvest of soybeans with 23% (d.b.) and drying with an air temperature below 100 °C had positive effects in maintaining the quality over the storage time, regardless of the storage condition. Among the changes that occurred, it was found that the storage time reduced the moisture content by an average of 1% (d.b.) at 15 and 23 °C (Tables 7 and 8). These changes occurred by variations of the relative humidity of the air (40 to 30%). In storage at 30 °C, the moisture content increased from 10 to 11% (d.b.) due to the relative humidity of the ambient air at 80%. According to Bischoff et al^41^, the grain storage at 30 °C causes excessive respiration, altering the physicochemical properties and losses in oil quality of approximately 59.6% (90 days), 67% (135 days), and 76% (180 days).

The most significant effects of soybean quality reduction were observed in paper packaging and a temperature of 30 °C. According to Maciel et al^42^ for a constant temperature, the equilibrium moisture hygroscopic content increases with the relative humidity. Although the temperature influences the hygroscopic equilibrium humidity, this influence is weak. This is because water is transferred from the air to the soybean when the relative humidity of the storage ambient air is higher than the equilibrium humidity^43^, being more intense when the soybeans are stored in high permeability packages (Tables 7 and 8).

The storage conditions at 15 and 23 °C in plastic bags were favorable for quality. The soybean storage in the temperature at 15 °C was favorable to the yield and the acidity index of the extracted oil, while the storage time was the main factor that altered the change in the acidity indexes. Mbofung et al^44^ reported increases in the soybean acid value for all storage conditions; however, increases in temperature and air humidity led to further grain deterioration^45^. Investigations according to evaluate the quality of the soybean grains stored in different conditions at 25 °C, the physicochemical properties, such as ash (4.7%), protein (3.9%), lipids (21.9%), and carbohydrates (34.4%) were not altered. Oppositely, at 35 °C, a reduction in the tegument color (88% to 85%) was observed, in addition to an increase in free fatty acids (3.7% to 4.7%) and, consequently, the grains acidity content due to the hydrolytic degradation of fat components by the action of lipase, in which these fatty acids are liberated from the triacylglycerol structures^18^. Assessing the effects of drying and storage on soybean quality, some studies found that the increase in grain drying temperature from 75 to 105 °C associated with storage conditions of 25 °C and 50%, 20 °C and 60%, 30 °C and 40% relative humidity over six months reduced the oil extraction yield and increased the acid index^46,47^.

Tables 7 and 8 were observed regardless of storage and packaging conditions, a significant reduction in the percentage of crude protein in the grains on the 8 months of storage. In the evaluation of the quality of soybeans stored for 6 months in permeable paper bags and polyethylene plastic bags at 3, 10, and 23 °C. Coradi et al^46^ found that the increased storage time reduced the quality of soybeans, regardless of storage conditions and packaging. In addition, the storage temperature of 23 °C was the most negatively altering the quality of soybeans. However, the storage in air temperature of 3 °C was most favorable for the quality of soybeans, although some quality results were similar, with storage at 10 °C.

As with other quality evaluations, it was observed that the crude protein content was higher in soybeans stored at lower temperatures. Lee & Cho^48^ evaluated soybean storage for 2 years, at room temperature, and observed a reduction in protein levels from 43 to 38.30%, for 1 and 2 years, respectively. Kibar^49^ and Rani et al^50^ studied soybean storage at different moisture contents (12 and 16% d.b.) and temperatures (8, 13, 18, 23, and 28 °C) and reported a reduction in crude protein content with increased moisture content and the temperature. Neethirajan et al^51^ found similar results, with a significant reduction in the soybean protein content at a storage temperature of 30 °C and relative humidity of 88%. Although the storage conditions affected the crude protein content in the soybean, storage at lower temperatures allowed greater conservation^44^.

Ziegler et al^52^ evaluated the effects of moisture content (12 and 15%) and storage temperature (11, 18, 25, and 32 °C) of soybeans on the functional properties of the protein isolate. Protein solubility reduced 18% with increasing temperature from 11 to 32 °C in soybean stored with 12% moisture. When the soybeans were stored with 15% moisture, the protein solubility reduced by 16% with increasing temperature from 11 to 32 °C. Furthermore, when soybeans were stored at the same temperature, for example, 25 °C, increasing moisture from 12 to 15% reduced protein solubility by 4%.

### Multivariate analysis

**Figure 4:**
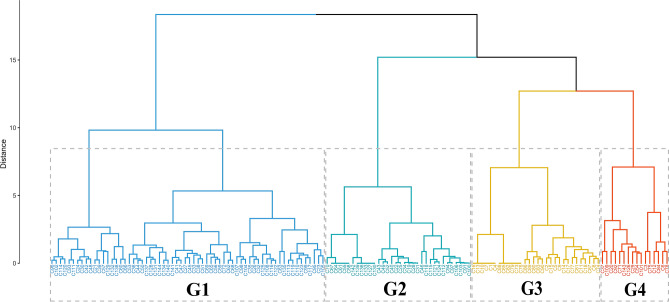
Cluster analysis of treatments using Euclidean distance and Ward's hierarchical method.

Cluster analysis showed the existence of four homogeneous groups for the variables evaluated (Fig. 4). G1 group gathered the largest number of treatments and stood out for the higher average of electrical conductivity and lower averages of acid oil and crude protein. The treatments allocated in this group belong to the higher storage times (4 and 8 months). G2 group allocated most treatments with zero storage time, which had the higher averages of oil yield and crude protein and intermediate values of electrical conductivity and acid oil. G3 and G4 groups allocated treatments from all storage times, and it is not possible to associate the grouping pattern to a specific storage period.

The treatments in the G3 group showed lower averages of electrical conductivity, in addition to intermediate values for the other variables. G4 group in turn brought together treatments with the higher averages of acid oil, in addition to intermediate values and with high variability for the other variables. The results indicated that there were effects of the association of the conditions of harvest, drying, and storage on the quality of the grains. It is important to highlight that storage time was the main study factor that impacted the groups formed^53^.

Similar results were observed by Ferreira et al^54^ evaluated the effects of drying temperature (30, 50, 70, 90, and 110 °C) and storage time (0 and 12 months) on physicochemical parameters in soybean. The authors reported that the increase in drying temperature resulted in a reduction in the quality of physical. In 12 months of storage, soybeans dried at 70, 90, and 110 °C showed higher (20, 65, and 14%, respectively) amounts of contamination than soybeans dried at 30 °C, accelerating the metabolism of grains, reducing antioxidant compounds such as isoflavones^54^, and reduces protein solubility and increases lipase activity and lipid acidity in soybeans^55^.

**Figure 5:**
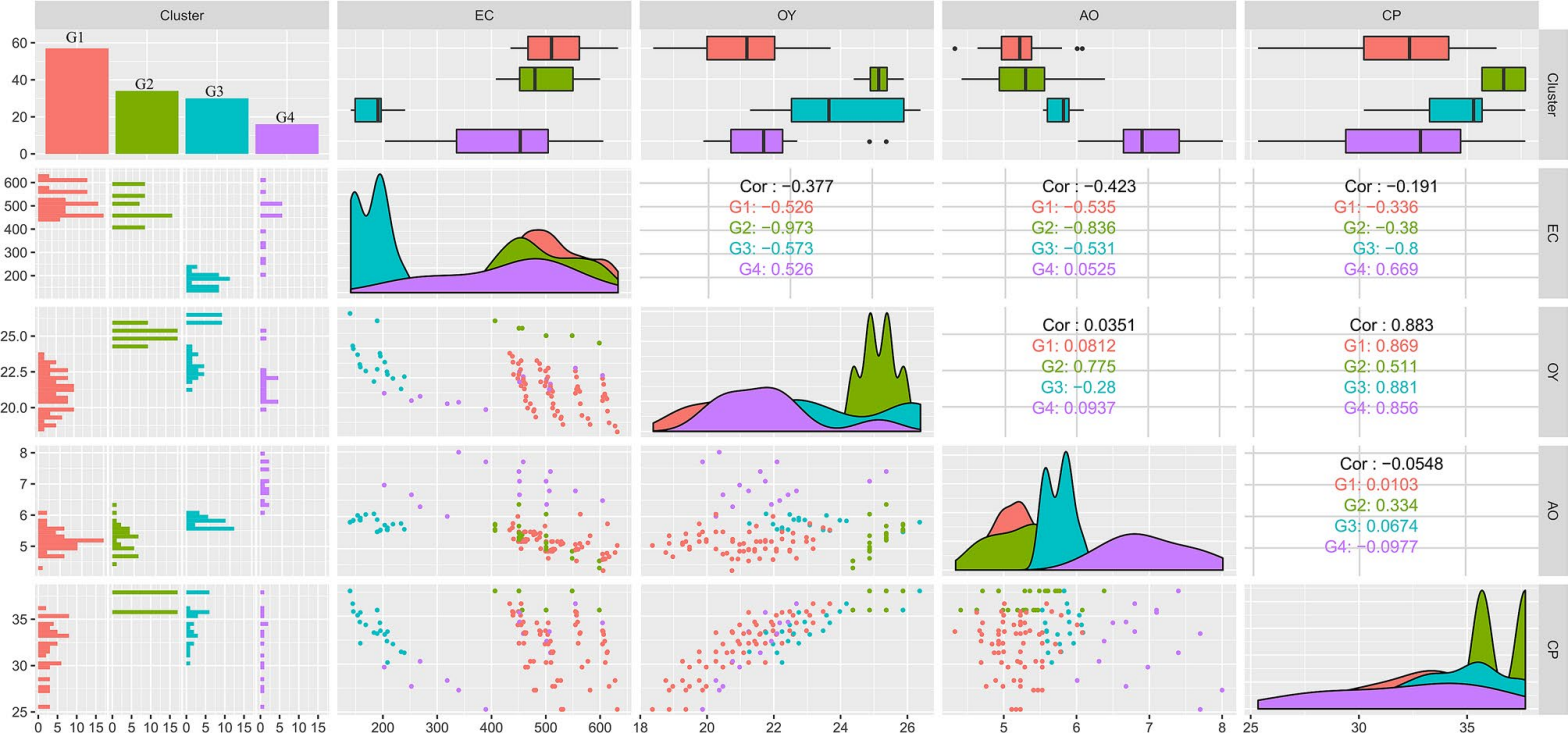
Dispersion and Pearson’s correlation between the variables evaluated according to the groups defined by the cluster analysis.

Regarding Pearson's correlations between the variables for each group, it is noted that the direction of the correlations was similar (Fig. 5). The electrical conductivity is negatively correlated and in low magnitude with all the physicochemical variables evaluated. However, it is to note that these correlations' magnitude was substantially more remarkable for the treatments allocated to the G2 group. These treatments also showed a positive and high magnitude correlation for acid oil and oil yield. Another correlation worth mentioning was that observed between crude protein and oil yield, which was positive and of high magnitude, regardless of the group formed.

Coradi et al^56^ verified changes in the yield of protein and oil extracted in the grains in function from the presence of fermented, rotten, and burned soybeans caused by the high drying temperature and storage conditions. Ramos et al^55^ found that the solubility of the protein isolates extracted from fermented, rotten, and burned soybeans are 17, 40, 59% lower compared to the protein isolate from not defective soybeans. The acidity of oil extracted from fermented, rotten, and burned soybeans is 969, 1350, 2248% higher than the acidity of oil extracted from not defective soybeans. Thus, the importance of optimizing the conditions for drying and storing soybeans is evident.

## Conclusions

The low drying air temperatures decreased the effective diffusivity and the time of volumetric shrinkage. Although storage time was the main factor influencing grain quality, the early harvest at 23% moisture content, adopting drying systems with air temperatures of 80 °C, and storage in controlled environments with temperatures below 23 °C are favorable to conserve the physicochemical quality of the soybean.

## Final considerations

The parameters obtained from soybean harvesting, drying, and storage make it possible to improve the management of the grain mass, to achieve better quality results. When applied at the farm level, it can enhance the production chain, improve transport and distribution logistics, reduce soybean losses, and add value to the marketing of soybeans. The results and conclusions obtained in this research are indicated for future investigations in soybean pre-processing and storage units, mainly at the farm level, to optimize harvest and post-harvest operations. For future research, it is suggested to carry out diagnoses on the different existing technologies of drying and storage, to propose a project that can more effectively implement the conclusive parameters of this study.

## Supplementary Information

Supplementary Table S1.
