# Supplementary material for: Mathematical modeling and multivariate analysis applied earliest soybean harvest associated drying and storage conditions and influences on physicochemical grain quality
Source: Sci Rep. 2021 Dec 2;11:23287. doi: 10.1038/s41598-021-02724-y (PMC8640013; doi:10.1038/s41598-021-02724-y)
Supplement: Supplementary file 1 — Supplementary Table S1. [file 41598_2021_2724_MOESM1_ESM.doc]

**Table S1.** Experimental design and clusters of drying and storage conditions

| Initial drying moisture content (%) | Drying air temperature (°C) | Packaging | Storage temperature (°C) | Storage time (months) | Treatments | Clusters |
| --- | --- | --- | --- | --- | --- | --- |
| 18 | Mixed | Permeable | 23 | 4 | C23 | G1 |
| 18 | Mixed | Permeable | 15 | 4 | C26 | G1 |
| 18 | Mixed | Impermeable | 23 | 4 | C32 | G1 |
| 18 | Mixed | Impermeable | 15 | 4 | C35 | G1 |
| 18 | 100 | Permeable | 30 | 4 | C38 | G1 |
| 18 | 100 | Permeable | 23 | 4 | C41 | G1 |
| 18 | 100 | Permeable | 15 | 4 | C44 | G1 |
| 18 | 100 | Impermeable | 30 | 4 | C47 | G1 |
| 18 | 100 | Impermeable | 23 | 4 | C50 | G1 |
| 18 | 100 | Impermeable | 15 | 4 | C53 | G1 |
| 18 | 120 | Permeable | 30 | 4 | C56 | G1 |
| 18 | 120 | Permeable | 23 | 4 | C59 | G1 |
| 18 | 120 | Permeable | 15 | 4 | C62 | G1 |
| 18 | 120 | Impermeable | 30 | 4 | C65 | G1 |
| 18 | 120 | Impermeable | 15 | 4 | C68 | G1 |
| 23 | Mixed | Permeable | 23 | 4 | C95 | G1 |
| 23 | Mixed | Permeable | 15 | 4 | C98 | G1 |
| 23 | Mixed | Impermeable | 23 | 4 | C104 | G1 |
| 23 | Mixed | Impermeable | 15 | 4 | C107 | G1 |
| 23 | 100 | Permeable | 30 | 4 | C110 | G1 |
| 23 | 100 | Permeable | 23 | 4 | C113 | G1 |
| 23 | 100 | Permeable | 15 | 4 | C116 | G1 |
| 23 | 100 | Impermeable | 30 | 4 | C119 | G1 |
| 23 | 100 | Impermeable | 23 | 4 | C122 | G1 |
| 23 | 100 | Impermeable | 15 | 4 | C125 | G1 |
| 23 | 120 | Permeable | 30 | 4 | C128 | G1 |
| 23 | 120 | Permeable | 23 | 4 | C131 | G1 |
| 23 | 120 | Permeable | 15 | 4 | C134 | G1 |
| 23 | 120 | Impermeable | 30 | 4 | C137 | G1 |
| 23 | 120 | Impermeable | 23 | 4 | C140 | G1 |
| 18 | Mixed | Permeable | 30 | 8 | C21 | G1 |
| 18 | Mixed | Permeable | 23 | 8 | C24 | G1 |
| 18 | Mixed | Permeable | 15 | 8 | C27 | G1 |
| 18 | Mixed | Impermeable | 30 | 8 | C30 | G1 |
| 18 | Mixed | Impermeable | 23 | 8 | C33 | G1 |
| 18 | Mixed | Impermeable | 15 | 8 | C36 | G1 |
| 18 | 100 | Permeable | 30 | 8 | C39 | G1 |
| 18 | 100 | Permeable | 23 | 8 | C42 | G1 |
| 18 | 100 | Permeable | 15 | 8 | C45 | G1 |
| 18 | 100 | Impermeable | 30 | 8 | C48 | G1 |
| 18 | 100 | Impermeable | 23 | 8 | C51 | G1 |
| 18 | 120 | Permeable | 30 | 8 | C57 | G1 |
| 18 | 120 | Permeable | 23 | 8 | C60 | G1 |
| 18 | 120 | Permeable | 15 | 8 | C63 | G1 |
| 18 | 120 | Impermeable | 30 | 8 | C66 | G1 |
| 18 | 120 | Impermeable | 23 | 8 | C69 | G1 |
| 18 | 120 | Impermeable | 15 | 8 | C72 | G1 |
| 25 | Mixed | Permeable | 30 | 8 | C93 | G1 |
| 25 | Mixed | Permeable | 23 | 8 | C96 | G1 |
| 25 | Mixed | Permeable | 15 | 8 | C99 | G1 |
| 25 | Mixed | Impermeable | 30 | 8 | C102 | G1 |
| 25 | Mixed | Impermeable | 23 | 8 | C105 | G1 |
| 25 | Mixed | Impermeable | 15 | 8 | C108 | G1 |
| 23 | 100 | Permeable | 30 | 8 | C111 | G1 |
| 23 | 100 | Permeable | 23 | 8 | C114 | G1 |
| 23 | 100 | Permeable | 15 | 8 | C117 | G1 |
| 23 | 100 | Impermeable | 30 | 8 | C120 | G1 |
| 23 | 100 | Impermeable | 23 | 8 | C123 | G1 |
| 23 | 120 | Permeable | 30 | 8 | C129 | G1 |
| 23 | 120 | Permeable | 23 | 8 | C132 | G1 |
| 23 | 120 | Permeable | 15 | 8 | C135 | G1 |
| 23 | 120 | Impermeable | 30 | 8 | C138 | G1 |
| 23 | 120 | Impermeable | 23 | 8 | C141 | G1 |
| 23 | 120 | Impermeable | 15 | 8 | C144 | G1 |
| 18 | Mixed | Permeable | 30 | 0 | C19 | G2 |
| 18 | Mixed | Permeable | 23 | 0 | C22 | G2 |
| 18 | Mixed | Permeable | 15 | 0 | C25 | G2 |
| 18 | Mixed | Impermeable | 30 | 0 | C28 | G2 |
| 18 | Mixed | Impermeable | 23 | 0 | C31 | G2 |
| 18 | Mixed | Impermeable | 15 | 0 | C34 | G2 |
| 18 | 100 | Permeable | 23 | 0 | C40 | G2 |
| 18 | 100 | Permeable | 15 | 0 | C43 | G2 |
| 18 | 100 | Impermeable | 30 | 0 | C46 | G2 |
| 18 | 100 | Impermeable | 23 | 0 | C49 | G2 |
| 18 | 100 | Impermeable | 15 | 0 | C52 | G2 |
| 18 | 120 | Permeable | 30 | 0 | C55 | G2 |
| 18 | 120 | Permeable | 23 | 0 | C58 | G2 |
| 18 | 120 | Permeable | 15 | 0 | C61 | G2 |
| 18 | 120 | Impermeable | 30 | 0 | C64 | G2 |
| 18 | 120 | Impermeable | 23 | 0 | C67 | G2 |
| 18 | 120 | Impermeable | 15 | 0 | C70 | G2 |
| 23 | Mixed | Permeable | 30 | 0 | C91 | G2 |
| 23 | Mixed | Permeable | 23 | 0 | C94 | G2 |
| 23 | Mixed | Permeable | 15 | 0 | C97 | G2 |
| 23 | Mixed | Impermeable | 30 | 0 | C100 | G2 |
| 23 | Mixed | Impermeable | 23 | 0 | C103 | G2 |
| 23 | Mixed | Impermeable | 15 | 0 | C106 | G2 |
| 23 | 100 | Permeable | 23 | 0 | C112 | G2 |
| 23 | 100 | Permeable | 15 | 0 | C115 | G2 |
| 23 | 100 | Impermeable | 30 | 0 | C118 | G2 |
| 23 | 100 | Impermeable | 23 | 0 | C121 | G2 |
| 23 | 100 | Impermeable | 15 | 0 | C124 | G2 |
| 23 | 120 | Permeable | 30 | 0 | C127 | G2 |
| 23 | 120 | Permeable | 23 | 0 | C130 | G2 |
| 23 | 120 | Permeable | 15 | 0 | C133 | G2 |
| 23 | 120 | Impermeable | 30 | 0 | C136 | G2 |
| 23 | 120 | Impermeable | 23 | 0 | C139 | G2 |
| 23 | 120 | Impermeable | 15 | 0 | C142 | G2 |
| 18 | 80 | Permeable | 30 | 0 | C1 | G3 |
| 18 | 80 | Permeable | 23 | 0 | C4 | G3 |
| 18 | 80 | Permeable | 15 | 0 | C7 | G3 |
| 18 | 80 | Impermeable | 30 | 0 | C10 | G3 |
| 18 | 80 | Impermeable | 23 | 0 | C13 | G3 |
| 18 | 80 | Impermeable | 15 | 0 | C16 | G3 |
| 23 | 80 | Permeable | 30 | 0 | C73 | G3 |
| 23 | 80 | Permeable | 23 | 0 | C76 | G3 |
| 23 | 80 | Permeable | 15 | 0 | C79 | G3 |
| 23 | 80 | Impermeable | 30 | 0 | C82 | G3 |
| 23 | 80 | Impermeable | 23 | 0 | C85 | G3 |
| 23 | 80 | Impermeable | 15 | 0 | C88 | G3 |
| 18 | 80 | Permeable | 30 | 4 | C2 | G3 |
| 18 | 80 | Permeable | 23 | 4 | C5 | G3 |
| 18 | 80 | Permeable | 15 | 4 | C8 | G3 |
| 18 | 80 | Impermeable | 30 | 4 | C11 | G3 |
| 18 | 80 | Impermeable | 23 | 4 | C14 | G3 |
| 18 | 80 | Impermeable | 15 | 4 | C17 | G3 |
| 23 | 80 | Permeable | 30 | 4 | C74 | G3 |
| 23 | 80 | Permeable | 23 | 4 | C77 | G3 |
| 23 | 80 | Permeable | 15 | 4 | C80 | G3 |
| 23 | 80 | Impermeable | 30 | 4 | C83 | G3 |
| 23 | 80 | Impermeable | 23 | 4 | C86 | G3 |
| 23 | 80 | Impermeable | 15 | 4 | C89 | G3 |
| 18 | 80 | Permeable | 15 | 8 | C9 | G3 |
| 18 | 80 | Impermeable | 23 | 8 | C15 | G3 |
| 18 | 80 | Impermeable | 15 | 8 | C18 | G3 |
| 23 | 80 | Permeable | 15 | 8 | C81 | G3 |
| 23 | 80 | Impermeable | 23 | 8 | C87 | G3 |
| 23 | 80 | Impermeable | 15 | 8 | C90 | G3 |
| 18 | 100 | Permeable | 30 | 0 | C37 | G4 |
| 23 | 100 | Permeable | 30 | 0 | C109 | G4 |
| 18 | Mixed | Permeable | 30 | 4 | C20 | G4 |
| 18 | Mixed | Impermeable | 30 | 4 | C29 | G4 |
| 18 | 120 | Impermeable | 15 | 4 | C71 | G4 |
| 23 | Mixed | Permeable | 30 | 4 | C92 | G4 |
| 23 | Mixed | Impermeable | 30 | 4 | C101 | G4 |
| 23 | 120 | Impermeable | 15 | 4 | C143 | G4 |
| 18 | 80 | Permeable | 30 | 8 | C3 | G4 |
| 18 | 80 | Permeable | 23 | 8 | C6 | G4 |
| 18 | 80 | Impermeable | 30 | 8 | C12 | G4 |
| 18 | 100 | Impermeable | 15 | 8 | C54 | G4 |
| 23 | 80 | Permeable | 30 | 8 | C75 | G4 |
| 23 | 80 | Permeable | 23 | 8 | C78 | G4 |
| 23 | 80 | Impermeable | 30 | 8 | C84 | G4 |
| 23 | 100 | Impermeable | 15 | 8 | C126 | G4 |

Permeable – Paper bags, Impermeable - Plastic raffia - polyethylene bags, Mixed – dried grains at 80, 100 and 120 °C and mixed for storage.
